# Changes in Cyclin E1 expression and *CCNE1* amplification in high-grade ovarian carcinomas post-PARP inhibitor exposure

**DOI:** 10.1038/s41416-026-03440-y

**Published:** 2026-05-05

**Authors:** Alexis Trecourt, Alexander Valent, Elisa Yaniz-Galende, Etienne Rouleau, Juan Francisco Grau Bejar, Félix Blanc-Durand, Elodie Edmond, Audrey Le Formal, Catherine Genestie, Alexandra Leary

**Affiliations:** 1 Hospices Civils de Lyon, Centre Hospitalier Lyon-Sud, Service de Pathologie Multi-Site, Lyon, France; 2 Centre pour l’innovation en cancérologie de Lyon (CICLY), Université Claude Bernard Lyon-1, Faculté de Médecine Lyon Sud, Lyon, France; 3 Institut National de la Santé et de la Recherche Médicale (INSERM) U981, Gustave Roussy, Université Paris-Saclay, Villejuif, France; 4 Gustave Roussy, Laboratoire de cytogénétique, Villejuif, France; 5 Gustave Roussy, Laboratoire de génétique des cancers, Villejuif, France; 6 Gustave Roussy, Service d’oncologie Médicale, Villejuif, France; 7 Gustave Roussy, Plateforme de pathologie expérimentale et translationnelle (PETRA), INSERM US23/CNRS UAR3655, Villejuif, France; 8 Gustave Roussy, Service de Pathologie, Villejuif, France

**Keywords:** Ovarian cancer, Ovarian cancer

## Abstract

**Background:**

Therapeutic options are limited for patients with high-grade ovarian carcinoma (HGOC) progressing after poly(adenosine diphosphate-ribose) polymerase-inhibitor (PARPi). WEE1/CDK2-inhibitors efficacy is under investigation in HGOC harbouring *CCNE1* amplification/Cyclin E1 overexpression. However, Cyclin E1 expression evolution after PARPi has not been studied. We aimed to describe Cyclin E1 expression/*CCNE1* copy number in post-PARPi HGOC samples and compare to paired samples from diagnosis and/or post-neoadjuvant chemotherapy (post-NACT).

**Methods:**

Thirty-eight patients with available post-PARPi samples were included; paired samples from diagnosis (*n* = 26) and/or post-NACT (*n* = 24) were collected. Cyclin E1 expression was quantified using immunohistochemistry (IHC). *CCNE1* copy number was evaluated using fluorescent in situ hybridisation (FISH).

**Results:**

Seventy-two percent (26/36) of HGOC were homologous recombination deficient. Intratumoral Cyclin E1 expression was homogenous in samples from synchronous but anatomically distinct tumour sites. However, Cyclin E1 expression increased significantly between diagnosis and progression post-PARPi (median H-score = 113 *versus* 163, respectively; *p* = 0.034). The proportion of Cyclin E1-high (H-score ≥ 150) tumours was 31% (8/26) at diagnosis, 42% (10/24) post-NACT, and increased significantly to 61% (23/38) post-PARPi (*versus* diagnosis; *p* = 0.024). In contrast, only 10% (2/20) of Cyclin E1-high HGOC exhibited *CCNE1* amplification ( ≥ 8 *CCNE1* copies).

**Conclusions:**

Cyclin E1 expression in HGOC increases at post-PARPi progression, independently of *CCNE1* amplification.

## Background

The advent of poly(adenosine diphosphate-ribose) polymerase inhibitors (PARPi) has changed the therapeutic landscape of homologous repair deficiency-positive (HRD + ) high-grade ovarian carcinomas (HGOC), significantly improving progression-free survival (PFS) and overall survival (OS) [1]. Cyclin E1 gene (*CCNE1*) amplification and/or Cyclin E1 protein (Cyclin E1) overexpression in high-grade serous ovarian carcinomas (HGSOC) have been associated with poor prognosis and resistance to platinum-based chemotherapy or PARPi [2, 3]. Furthermore, in HGOC, *CCNE1* amplification was initially reported as mutually exclusive with HRD+ status [4, 5].

However, double classifier tumours harbouring both HRD and *CCNE1* amplification have been recently reported, representing between 6 to 20% of HGOC [6–8]. These double classifiers were associated with a poorer OS as compared to HRD + HGOC without *CCNE1* amplification, and similar to that of homologous repair proficient (HRP) HGOC with *CCNE1* amplification [7]. Lheureux et al. reported *CCNE1* amplification in some *BRCA1/2*-mutated HGSOC following multiple lines of treatment (median five prior lines) [9]; in this report, *CCNE1* amplification was the second most common genomic alteration (16%) in ovarian HGSOC at post-PARPi progression after reversion mutations in homologous recombination genes (19%) [9]. These findings suggest that *CCNE1* amplification status may change between diagnosis and relapse depending on intervening systemic therapies [8]. Patients progressing post-PARPi have poor prognosis, are often chemo-resistant, and new therapies are thus crucially needed [10]. Novel approaches targeting the cell cycle control and replication stress are under investigation in *CCNE1*-amplified/Cyclin E1-overexpressed HGOC. These include WEE1 G2 checkpoint kinase (Wee1)-inhibitors, cyclin-dependent kinase 2 (CDK2)-inhibitors, and protein kinase membrane-associated tyrosine/threonine 1 (PKMYT1)-inhibitors [4, 11–15]. Recent clinical trials enrolled patients according to both Cyclin E1-overexpression and *CCNE1*-amplification [16, 17], or according to Cyclin E1 overexpression regardless of *CCNE1* amplification [15]. In the latter, the overall response rate of patients with recurrent platinum resistant HGSOC treated with *adavosertib* was 53%, suggesting that Cyclin E1 immunohistochemistry (IHC) could predict response to this class of agents. Indeed, a subset of *CCNE1* non-amplified HGOC overexpresses Cyclin E1 [2, 3]. However, the variation of Cyclin E1 overexpression with treatment lines, and especially after PARPi, has been understudied.

The primary objective of this study was to evaluate Cyclin E1 expression by IHC, at progression during or following PARPi therapy, in a cohort of HGOC patients enriched for HRD+ tumours, where the prevalence of *CCNE1* amplification is expected to be low, and to compare this expression to that of earlier time-points (at diagnosis and after neoadjuvant chemotherapy [post-NACT]). The secondary objectives were: i) to determine whether Cyclin E1 overexpression could be attributed to *CCNE1* amplification or other numerical or structural alterations of *CCNE1* locus and/or chromosome 19 using fluorescent in situ hybridisation (FISH); and ii) to assess the temporal heterogeneity of *CCNE1* amplification status between the different time-points.

## Methods

### Study cohort, study design, and collection of clinical and laboratory data

All patients with a diagnosis of HGOC, who relapse during or following PARPi were identified from our local database (*Gustave Roussy*, Paris, France) using REDCap software (Nashville, TN, United States of America [USA]). Only patients with available formalin-fixed and paraffin-embedded (FFPE) samples from the relapse during or following PARPi were included. For these patients, when available, samples from different time-points (from diagnosis and following NACT) were included. Samples were then divided in three different categories, according to the time-points: (i) samples from the diagnosis or samples from up-front debulking surgeries in the absence of neoadjuvant chemotherapy (diagnostic samples); (ii) samples from interval debulking surgeries after NACT (post-NACT samples); and (iii) samples from the relapse during or following PARPi (post-PARPi samples). Clinical data and follow-up were retrospectively collected until May 2025.

### HRD status assessment

The determination of HRD status and its mechanism was carried out as part of the routine clinical practice and not for the purpose of the present study.

#### DNA extraction and targeted-next-generation sequencing (NGS)

For each patient, tumour genomic DNA was extracted from FFPE tissues using QIAsymphony DSP DNA Kits (Qiagen, Hilden, Germany), according to the manufacturer’s instructions.

Libraries were generated using SureSelect XT HS Target Enrichment System (Agilent Technologies, Santa-Clara, CA, USA) and were sequenced on a NextSeq 500 sequencer (Illumina, San Diego, CA, USA). The NGS panel includes the homologous recombination repair genes: *BRCA1* (NM_007294.3; exons 2 to 3 and 5 to 24), *BRCA2* (NM_000059.3; exons 2 to 27), *RAD51C* (NM_058216.2; exon 1 to 9), *RAD51D* (NM_001164269.1; exon 2 to 10), *CCNE1* (NM_001238.3; exons 2 to 12), *BRIP1* (NM_032043.2; exons 2 to 20), *BARD1* (NM_000465.3; exons 1 to 11), *CHEK2* (NM_007194.3; exons 2 to 15), *PALB2* (NM_024675.3; exons 1 to 13), *TP53* (NM_000546.5; exon 1 to 11), *CDK12* (NM_016507.3; exons 1 to 14), and *ATM* (NM_000051.3; exons 2 to 63).

#### HRD status assessment for tumours in the absence of *BRCA1/2* pathogenic variant

The HRD status was determined either: i) using FFPE tissue tested with the MyChoice^®^ CDx panel (Myriad Genetics, Salt Lake City, UT, USA), as previously described [18], the genomic instability score (GIS) cutoff to defined instability was ≥ 42; or ii) using shallow whole genome sequencing (sWGS) to assess genome-wide copy number alterations (CNAs) GIS, as previously described [19, 20]. Briefly for sWGS, library was prepared using SureSelectXTHS or SureSelectXTHS2 kit (Agilent Technologies) and sequenced on NextSeq (Illumina). Tumour purity and large-scale CNAs were estimated using the ichorCNA algorithm (v0.3.2), which is optimised for low pass sequencing data and enables estimation of tumour fraction and identification of broad copy number alterations. GIS was calculated by a shallowHRD software which integrates CNA profiles. Thresholds to classify genomic instability scores (GIS) were as follows: ≥20 defining high-GIS, <15 for low-GIS, and 15–20 for intermediate-GIS.

#### Classification in HRD/HRP status

Using results from molecular and epigenetics analyses, HGOC were classified as: (i) HRD+ related to pathogenic *BRCA1* or *BRCA2* variant (HRD + /*BRCA1/2*); (ii) HRD+ non-related to pathogenic *BRCA1* or *BRCA2* variant (HRD + /non-*BRCA1/2*); (iii) HRP (HRD-negative); and iv) results unknown/not available (NA).

### Cyclin E1 IHC

For all samples included and according to the manufacturer’s instructions, an anti-Cyclin E1 IHC (clone EP126, ref. AC-0120RUO, Cell Marque, Rocklin, CA, USA) was performed, using a Ventana Discovery Ultra automated immunostainer (Roche Diagnostics, Rotkreuz, Switzerland). Detailed IHC method is presented in Supplementary Material 1. The nuclear Cyclin E1 expression was quantified in tumour cells by two surgical pathologists (A.T. and C.G.). An H-score (from 0–300) was calculated, and the threshold ≥ 150 was chosen to define overexpression (Cyclin E1-high *versus* Cyclin E1-low). Positive and negative controls were used for each run (Supplementary Fig. 1).

Of note, for six tumours, the IHC was performed on two different paraffin blocks of the same tumour and the H-scores obtained were compared, in order to investigate the intratumoral (spatial) heterogeneity of Cyclin E1 expression.

### *CCNE1* FISH

*CCNE1* FISH was performed on post-PARPi samples to investigate whether the Cyclin E1 overexpression using IHC was due to *CCNE1* amplification or other numerical or structural alteration. FISH were also performed on diagnostic and post-NACT samples to study temporal heterogeneity of *CCNE1* copy number according to treatments. FISH were performed on whole tissue sections, using a *CCNE1*-centromere chromosome 19 (CEN19) FISH probe (Empire Genomics, Buffalo, NY, USA), and the ZytoLight FISH-Tissue Implementation Kit (ZytoVision, Bremerhaven, Germany). Detailed FISH method is presented in Supplementary Material 1.

The interpretation was performed by a surgical pathologist (A.T.) and a cytogeneticist (A.V.). The FISH signals were counted under 100 × objective lens. *CCNE1* amplification was defined as ≥ 8 copies of *CCNE1* per tumour cell. In case of *CCNE1* amplification, the spatial distribution of the signals was also noted (structural alterations), either as double minute-type amplicon (discrete, countable signals), or as homogenously staining region (HSR)-type amplicon (aggregated clusters of signals), or ring-like structures suggesting an amplification within a ring chromosome. Moreover, the intratumoral homogeneity/heterogeneity of the amplification was specified (presence/absence of different clones). In the absence of *CCNE1* amplification, the tumours were classified in four other different patterns: i) *CCNE1* gain (*CCNE1*/CEN19 ratio ≥ 2 but *CCNE1* copy number being < 8); ii) high polysomy of chromosome 19 (≥4 copies of *CCNE1* and CEN19 signals in > 40% of cells); iii) polyploidization (high polyploidy of the whole genome in giant tumour cells with pleomorphic features observed on the hematoxylin-eosin-stained slide, after taxane-based chemotherapy); and iv) absence of *CCNE1* significant alteration (diploidy, triploidy, or tetraploidy in less than 40% of tumour cell). In the absence of *CCNE1* significant alteration, CEN19 alteration (gain or amplification) were also noted. Positive and negative controls were used (Supplementary Fig. 2).

Of note, to confirm the polyploidization phenomenon, a *JAZF1* probe (targeting the chromosome 7; ZytoVision) was used. The same technical conditions and interpretation criteria were used.

### Statistics

Descriptive statistics were used to analyse clinical, immunohistochemical, and molecular data. Dichotomous variables were expressed as counts and proportions and continuous variables were expressed as medians and ranges.

A Fisher’s exact test and Mann–Whitney test were used to compare immunohistochemical and molecular results between the three different samples groups (unpaired). To allow stronger statistical comparison and matched-pair analysis, the Cyclin E1 H-score of diagnostic and post-NACT samples were combined. For patients with both diagnostic and post-NACT samples, the mean H-score of the two values was used and then compared to the post-PARPi H-score using Wilcoxon statistical test.

The correlation between mean *CCNE1* copy number per tumour cell and Cyclin E1 H-score for diagnostic samples was calculated using Pearson correlation.

Survival analyses were performed using the Kaplan–Meier method and log-rank test to compare the median OS and the median PFS of Cyclin E1-high and low tumours. These survival analyses were performed using IHC findings from the post-PARPi samples, to investigate whether the Cyclin E1 overexpression could have a prognostic value at the time of the post-PARPi progression, for patients treated with platinum-based chemotherapy. Median PFS post-PARPi (defined as the time between the beginning of the post-PARPi progression treatment and the next relapse) and the median OS post-PARPi (defined as the time between the beginning of the post-PARPi progression treatment and the date of last news or death) were calculated. In addition, in this group of patients for whom the post-PARPi progression was treated with platinum-based chemotherapy, stratified survival analyses (PFS) were also performed to study if other criteria, such as the HRD status and the number of prior chemotherapy lines (1 or > 1), could also affect the survival.

All statistical analyses were performed using GraphPad (v.10.0.2) and a *p*-value < 0.05 was considered statistically significant.

## Results

### Patients and tumour samples

A total of 88 samples from 38 patients were collected, including 26 diagnostic samples (12 biopsies, 14 surgical specimens), 24 post-NACT samples (24 surgical specimens), and 38 post-PARPi samples (27 biopsies, 11 surgical specimens).

**Fig. 1:**
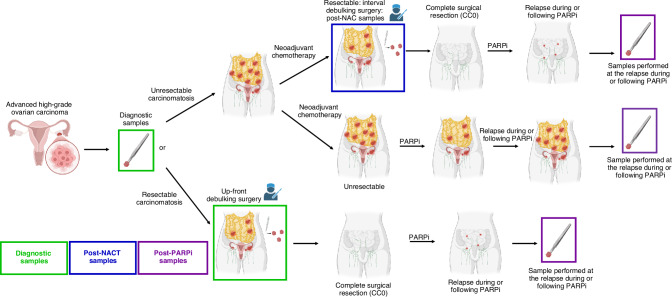
Study design and inclusion of samples. Samples included herein were divided in three different groups, according to the sampling time-points: (i) samples from the diagnosis or samples from up-front debulking surgeries in the absence of neoadjuvant chemotherapy (diagnostic samples; green); (ii) samples from interval debulking surgeries after neoadjuvant chemotherapy (post-NACT samples; blue); and (iii) samples from the relapse during or following PARPi (post-PARPi samples; purple). NACT: Neoadjuvant chemotherapy; PARPi: Poly(adenosine diphosphate-ribose) polymerase inhibitor.

The median age at diagnosis was 60.5 years old (range: 25–70). The international federation of gynaecologist and obstetrics (FIGO) stage at diagnosis was IIIB in 3/38 (7.9%) patients, IIIC for 29/38 (76.3%), and IVB for 6/38 (15.8%). Initially, patients were mainly treated with NACT (27/38, 71.1%) followed by interval debulking surgery in 24/27 (88.9%) patients, and 11/38 (28.9%) patients benefited from up-front debulking surgery and then adjuvant platinum-based chemotherapy. PARPi were prescribed as first-line maintenance therapy in 27/38 (71.1%) patients and as maintenance therapy after a platinum-sensitive relapse in 11/38 (28.9%). The mean and median number of prior platinum-based chemotherapy lines were 1.5 and 1, respectively (ranges:1–5). The post-PARPi progression occurred during PARPi maintenance therapy in 24/38 (63.2%) patients and following PARPi in 14/38 (36.8%). The post-PARPi progression was treated using platinum-based chemotherapy for 28/38 (73.7%) patients. There was no difference in the platinum regiment used. The majority of HGOC were HRD+ (72%, 26/36) including 17/36 (47.2%) tumours classified as HRD+ related to pathogenic *BRCA1* or *BRCA2* variant (HRD + /*BRCA1/2*), 9/36 (25%) as HRD+ non-related to pathogenic *BRCA1* or *BRCA2* variant (HRD + /non-*BRCA1/2*), 10/36 (27.8%) as HRP (HRD-negative; Table 1), and HRD status unknown for 2/38 (5.3%) HGOC due to poor DNA quality. Sample distribution among groups and clinical data are presented in Fig. 1.

**Table 1:** Characteristics of the cohort.

Clinical data, Sampling, and histopathological and molecular analyses	
Total number of patients included, *n*	38
Median age at diagnosis of HGOC, year (ranges)	60.5 (25–70)
FIGO stage at diagnosis, % (n/N)	
IIIB	7.9 (3/38)
IIIC	76.3 (29/38)
IVB	15.8 (6/38)
Histopathological subtype of tumours, % (n/N)	
HGSOC	89.5 (34/38)
EC	7.9 (3/38)
CCC	2.6 (1/38)
Molecular status, % (n/N)	
HRD + /*BRCA1/2*	47.2 (17/36)
*BRCA1*-mutated	76.5 (13/17)
*BRCA2*-mutated	23.5 (4/17)
HRD + /non-*BRCA1/2*	25 (9/36)
HRP	27.8 (10/36)
NA	5.3 (2/38)
Up-front debulking surgery, % (n/N)	28.9 (11/38)
Neoadjuvant platinum-based chemotherapy, % (n/N)	71.1 (27/38)
Followed by interval debulking surgery	88.9 (24/27)
Not eligible to interval debulking surgery	11.1 (3/27)
CC-score of patents after interval debulking surgery, % (n/N)	
CC-0	95.8 (23/24)
CC-2	4.2 (1/24)
Median duration of PARPi treatment, month (ranges)	23.5 (1–48)
Relapse during or following PARPi, % (n/N)	
During PARPi	63.2 (24/38)
Following PARPi	36.8 (14/38)
Treatment following PARPi, % (n/N)	
Platinum-based chemotherapy	73.7 (28/38)
Other	26.3 (10/38)
Death from disease, % (n/N)	47.3 (18/38)
Median PFS (first relapse), month (ranges)	31 (14–97)
Median OS, month (ranges)	71.5 (22–249)
Proportion of patients who relapsed again after the treatment of the post-PARPi progression, % (n/N)	65.8 (25/38)
Median specific PFS post-PARPi, month (ranges)	12 (2–41)
Median specific OS post-PARPi, month (ranges)	28.5 (2–75)

*CCC* Clear-cell carcinoma, *CC-score* Completeness of cytoreduction score, *EC* Endometrioid carcinoma, *FIGO* International federation of gynaecologist and obstetrics, *HGSOC* High-grade serous ovarian carcinoma, *HRD* Homologous repair deficiency, *HRP* Homologous repair proficiency, *N* Total number, *n* Number, *NA* Not available, *NACT* Neoadjuvant chemotherapy, *OS* Overall survival, *PARPi* Poly(adenosine diphosphate-ribose) polymerase inhibitor, *PFS* Progression-free survival.

Of note, other pathogenic variants identified by NGS analysis as part of the routine clinical practice included mutations of *TP53* in 32/36 (88.9%) tumours, *ATM* in 2/36 (5.6%), and *CDK12* in 1/36 (2.8%). As expected in this cohort enriched in HRD+ tumours, *CCNE1* amplifications were rare at diagnosis, identified in only one HGOC using targeted-NGS. Clinical and histopathological data, and molecular status are detailed in Table 1.

### Cyclin E1 expression and correlation clinical outcome

**Fig. 2:**
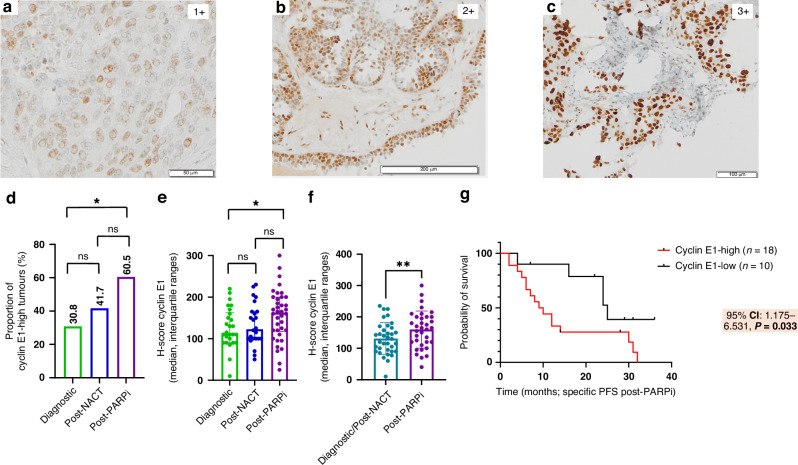
Increase Cyclin E1 expression from diagnosis to the post-PARPi progression. **a**–**c** Cyclin E1 immunohistochemistry showing mainly 1 + , 2 + , and 3 + intensity expression, respectively (x200 magnification). **d** The number of Cyclin E1-high tumours in post-PARPi samples was significantly higher than that of diagnostic samples (*p *= 0.024). **e** The median Cyclin E1 H-score was significantly higher in post-PARPi samples *versus* diagnostic samples (*p* = 0.034). **f** When combining the H-score of diagnostic/post-NACT samples to compare it to that of post-PARPi samples, a significant difference was found (*p* = 0.0051). **g** Concerning patients treated using platinum-based therapy at the post-PARPi progression, the median PFS post-PARPi was significantly better when the H-score was < 150 compared to ≥ 150 on post-PARPi samples (25 *versus* 9.5 months; 95% CI: 1.175–6.531; *p* = 0.033). CI: Confidence interval; NACT: Neoadjuvant chemotherapy; PARPi: Poly(adenosine diphosphate-ribose) polymerase inhibitor; PFS: Progression-free survival. *p < 0.05.

Firstly, to evaluate intratumoral heterogeneity in Cyclin E1 expression, Cyclin E1 IHC was performed on different paraffin blocks from two synchronous but anatomically distinct tumour sites for six patients. The median H-score difference between paraffin blocks was low, evaluated at 10.8 (ranges: 5–20). However, we observed a temporal heterogeneity in Cyclin E1 expression with an increase over time. Indeed, the median H-score for Cyclin E1 expression was 113 (ranges: 10–220) for diagnostic samples, 123 (ranges: 50–230) for post-NACT samples, and 163 (ranges: 25–300) for post-PARPi samples. Post-PARPi samples had a statistically significant higher H-score compared to diagnostic samples (*p* = 0.034), while the difference between diagnostic and post-NACT samples (*p* = 0.633), and post-NACT and post-PARPi samples (*p* = 0.127) was not statistically significant (Fig. 2). As no statistical difference was found between Cyclin E1 H-score of diagnostic and post-NACT samples, the H-score of both groups were combined and compared to that of the post-PARPi samples. Thirty-five paired samples were available for this analysis; a significant statistical difference was found between diagnostic/post-NACT samples (median H-score: 117.5 [ranges: 10–235]) *versus* post-PARPi samples (median H-score: 165 [ranges: 40–300]) (*p* = 0.0051). Moreover, using a H-score cut-off of ≥ 150 to define overexpression, the percentage of Cyclin E1-high tumours was 31% (8/26) at diagnosis, 42% (10/24) post-NACT, and 61% (23/38) post-PARPi. The proportion of Cyclin E1-high tumours post-PARPi was significantly higher compared to diagnostic samples (*p* = 0.024), while the differences between diagnostic and post-NACT samples (*p* = 0.557) and between post-NACT and post-PARPi samples (*p *= 0.194) were not statistically significant. Interestingly, among the 18 patients with Cyclin E1-low tumours at diagnosis, 33.3% (6/18) had Cyclin E1-high tumours post-PARPi. In contrast, none of the eight Cyclin E1-high tumours at diagnosis converted to Cyclin-E1-low post-PARPi. There was no significant difference in the H-score of tumours from patients who relapsed during *versus* following PARPi (median H-score: 170 *versus* 160, respectively; *p* = 0.595).

As Cyclin E1 overexpression has been associated with poor platinum response, we then evaluated outcomes for patients treated with platinum at the progression post-PARPi, according to the Cyclin E1 expression. Twenty-eight patients received platinum-based chemotherapy after-PARPi progression. Median PFS under platinum post-PARPi was significantly shorter for patients with Cyclin E1-high tumours compared to Cyclin E1-low tumours (9.5 *versus* 25 months, respectively; 95% CI: 1.175–6.531; *p* = 0.033). In contrast, in this group of 28 patients, no significant difference in survival (PFS) or major modifications in the Kaplan–Meier curve shapes were observed compared to Fig. 2g, when performing stratified survival analyses according to the HRD status or the number of prior chemotherapy line(s) before PARPi therapy (Supplementary Fig. 3).

### Numerical and structural changes of *CCNE1* in Cyclin E1-high HGOC and correlation between FISH and IHC results

Given that only one tumour was found to be *CCNE1* amplified by NGS at diagnosis, we aimed to evaluate whether other numerical or structural changes in *CCNE1* could account for protein overexpression using FISH, which allows a spatial evaluation of *CCNE1* locus-specific changes.

**Fig. 3:**
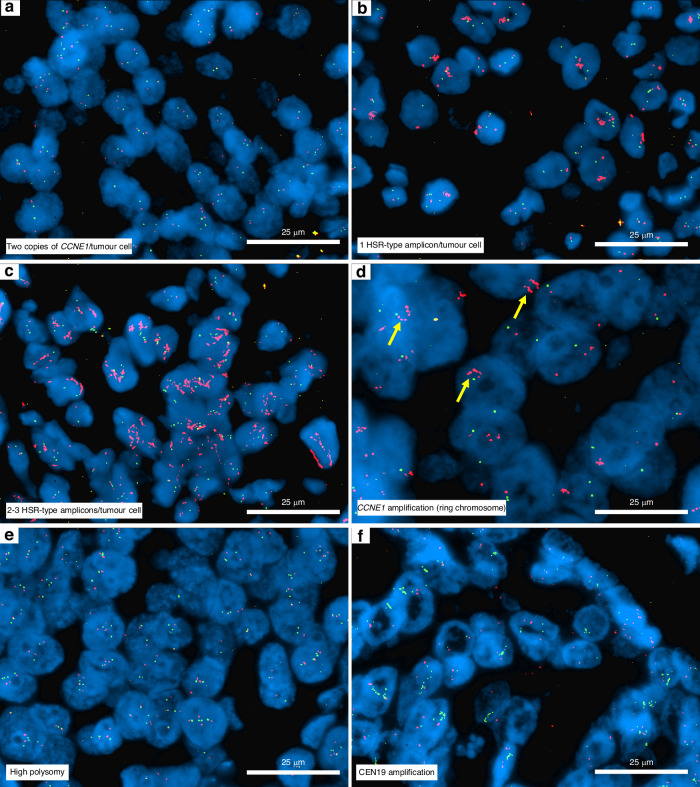
*CCNE1* FISH results (red spots: *CCNE1*; green spots: CEN19). **a** *CCNE1* FISH: two copies of *CCNE1* per tumour cell (x1000 magnification). **b** *CCNE1* amplification (one HSR-type amplicon per tumour cell; x1000 magnification). **c** *CCNE1* amplification (two to three HSR-type amplicons per tumour cell; x1000 magnification). **d** *CCNE1* amplification (spatial distribution suggesting a ring chromosome [yellow arrows]; x1000 magnification). **e** High polysomy (x1000 magnification). **f** Amplification of the CEN19 region (x1000 magnification). CEN19: Centromere chromosome 19; FISH: Fluorescent in situ hybridisation; HSR: homogenously staining region.

Seven of 88 (8%) samples failed FISH due to technical issues (three post-NACT samples and four post-PARPi samples). Thirty-four post-PARPi samples had both IHC and FISH results available. Among them, 20/34 (58.8%) were Cyclin E1-high and 14/34 (41.2%) were Cyclin E1-low. Using a threshold of ≥ 8 copy number of *CCNE1* to define amplification, only 10% (2/20) of Cyclin E1-high HGOC exhibited *CCNE1* locus amplification by FISH. For these two cases, the structural alteration of *CCNE1* amplification was an HSR-type amplicon in one case (HRP status; this *CCNE1* amplification was also identified using targeted-NGS), while ring-like structures suggesting ring chromosomes were identified for the other (HRD + /non-*BRCA1/2* status; the NGS analysis was not feasible for this tumour; Fig. 3). In other Cyclin E1-high HGOC, without *CCNE1* amplification, we observed a *CCNE1* gain (*i.e. CCNE1*/CEN19 ratio ≥ 2 but *CCNE1* copy number being < 8) in 2/20 (10%) tumours (one HRP status and one HRD + /non-*BRCA1/2*), and a high polysomy in 7/20 (35%) tumours (three HRP status, three HRD + /*BRCA1/2*, and one HRD + /non-*BRCA1/2*). However, for 9/20 (45%), neither *CCNE1* amplification, gain, nor high polysomy were observed; within these nine tumours, one harboured a gain of the CEN19. For the remaining 8/20 (40%) Cyclin E1-high tumours post-PARPi, no changes in *CCNE1* nor CEN19 were found (Table 2). Only one Cyclin E1-low tumour harboured high-polysomy, and no gain nor amplification were found in this subgroup. For diagnostic samples, the correlation between Cyclin E1 H-score and mean *CCNE1* copy number per tumour cell was moderate only (*r *= 0.42) (Supplementary Fig. 4).

**Table 2:** FISH findings in Cyclin E1-high HGOC post-PARPi therapy.

*CCNE1* and/or CEN19 numerical and structural alteration	Proportion of Cyclin E1-high tumours, % (n/N)
*CCNE1* amplification	10 (2/20)^a^
*CCNE1* gain	10 (2/20)
High-polysomy of chromosome 19	35 (7/20)
CEN19 gain without *CCNE1* alteration	5 (1/20)
No significant alteration of *CCNE1* and CEN19	40 (8/20)

*CEN19* Centromere chromosome 19, *FISH* Fluorescent in situ hybridisation, *HGOC* High-grade ovarian carcinomas, *HSR* homogenously staining region, *N* Total number, *n* Number, *PARPi* poly(adenosine diphosphate-ribose) polymerase-inhibitor.

^a^one HSR-type amplicon and one amplification with ring-like structures.

We observed intratumoral heterogeneity in the *CCNE1* copy number in 3/81 (3.7%) tumours. Two tumours were composed of one clone harbouring a *CCNE1* gain and another without gain, and one *CCNE1*-amplified tumour was composed of a clone harbouring one HSR-type amplicon per cell and another clone harbouring three HSR-type amplicons per cell. These three heterogeneous cases were post-PARPi samples.

We also observed a temporal variation of the *CCNE1* copy number in 2/33 (6.1%) patients: one loss of *CCNE1* amplification and one increase in the number of HSR-type amplicons per cell, both in post-PARPi setting. Only one clear cell carcinoma was included in the present study; this case exhibited a *CCNE1* double-minute-type amplicon in the diagnostic sample and an HSR-type amplicon in the post-PARPi sample.

**Fig. 4:**
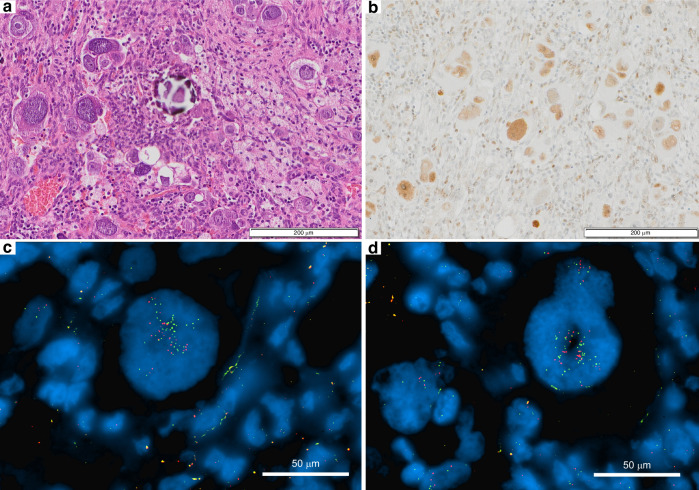
Polyploizidation phenomenon after neoadjuvant taxane-based chemotherapy. **a** HES-stained slide showing giant pleomorphic tumour cells (x100 magnification). **b** Cyclin E1 immunohistochemistry showing 1+ and 2+ expression intensity within the giant pleomorphic tumour cells (x100 magnification). **c**, **d** FISH showing > 20 copies of *CCNE1* and CEN19 per tumour cell after taxane-based chemotherapy, suggesting a polyploizidation phenomenon. FISH: Fluorescent in situ hybridisation; HES: Hematoxylin-eosin-saffron.

Finally, a polyploidization phenomenon (i.e., high polyploidy in giant tumour cells with pleomorphic morphological features after taxane-based chemotherapy) was detected in 3/21 (14.3%) post-NACT samples (Fig. 4 and Supplementary Fig. 5). The polyploidization phenomenon was systematically associated with a H-score > 150 post-NACT. However, for one of these cases, the H-score was < 150 at both diagnosis and post-PARPi, and no change in *CCNE1*/CEN19 and was observed at these time-points (*i.e*. interpreted as artifactual/“false positive” H-score > 150 for the post-NACT sample).

## Discussion

Most data regarding the prevalence and prognostic value of *CCNE1* amplification and Cyclin E1 overexpression were generated in treatment-naive tumour samples from unselected patients with HGOC [2, 3, 21]. In the present study, we aimed to evaluate Cyclin E1 expression in HGOC post-PARPi, and to compare this expression to that at diagnosis and post-NACT. Over 60% of post-PARPi samples exhibited an overexpression of Cyclin E1 (H-score > 150), in the absence of *CCNE1* amplification in most tumours (90%). Moreover, Cyclin E1 expression increased over time, with both Cyclin E1 median H-score and proportion of Cyclin E1-high tumours being significantly higher at post-PARPi progression compared to diagnosis. In contrast, *CCNE1* amplification status showed little variation with time. Finally, Cyclin E1 overexpression was significantly associated with poorer outcomes in patients receiving platinum-based chemotherapy following post-PARPi progression, although these results should be validated on larger cohort of patients.

In the present study, we observed a lower prevalence of *CCNE1* amplification (5.9%, 2/34 post-PARPi samples) than previously reported in HGSOC ( ~ 20%) [21]. This is likely explained by the enrichment in HRD+ tumours with an expected lower prevalence of *CCNE1* amplification [4, 5]. Indeed, only one HRD+ tumour herein exhibited *CCNE1* amplification using FISH and was an HRD + /non-*BRCA1/2*, as expected [6–8]. In addition, we used a stringent threshold of ≥ 8 copy number of *CCNE1* to define amplification, as previously reported using FISH or other morphological molecular techniques [2, 3, 15]; criterion often recommended for cytogenetic definition of gene amplification [22].

Given the low prevalence of *CCNE1* amplification in our cohort, we sought to investigate whether other numerical or structural changes in *CCNE1* could explain Cyclin E1 increased expression in post-PARPi samples. Using FISH, *CCNE1* gains or high polysomy were observed in 45% of Cyclin E1-high post-PARPi samples, whereas only one Cyclin E1-low tumour harboured high-polysomy. These alterations can only be identified using a morphological molecular technique, such as FISH, by counting both *CCNE1* and CEN19 signals, unlike NGS where results are influenced by tumour cell ploidy and are less reliable for detecting *CCNE1* gains (between 2–7 copies) [2, 23]. However, for 45% of Cyclin E1-high tumours, we found no *CCNE1* amplification, *CCNE1* gain, nor high-polysomy. In these tumours, one hypothesis is that the overexpression could be explained by other factors such as: (i) inactivating pathogenic variants of *FBXW7*, which encodes F-box protein FBXW7 ubiquitinase, an endogenous degrader of Cyclin E1 [24]; (ii) upregulation of the activity of the CCNE1-CDK2 protein complex by the inactivation of kinase-inhibitory protein/CDK-interacting protein (KIP/CIP)-cyclin-dependent kinase inhibitors [25]; or (iii) the increase transcriptional activity of *CCNE1*, although *CCNE1* mRNA quantification does not appear to be relevant for clinical purposes (*i.e*., not correlated to poor OS, and the correlation reported was weak to moderate only between *CCNE1* mRNA expression level and *CCNE1* amplification or Cyclin E1 overexpression, when using Pearson correlation) [2, 3]. However, these hypotheses were not tested herein.

Currently, patients progressing during or after PARPi therapy represent a major unmet clinical need, as their response to subsequent chemotherapy is limited [26]. Novel strategies targeting cell cycle regulation or replication stress are currently being investigated in this setting including CDK2 inhibitors, Wee-1 inhibitors, or PKMYT1 inhibitors in Cyclin E1-high HGSOC [3, 4, 11–15, 27]. In the present cohort, one third of the Cyclin E1-low HGOC at diagnosis converted to Cyclin E1-high post-PARPi progression. It is worth noting that we used the EP126 antibody clone, which is one of the most reported for the investigation of Cyclin E1 expression in ovarian and endometrial carcinomas [28]. Moreover, this clone was reported as the one allowing the best staining, in a study comparing 6 different Cyclin E1 IHC clones [2]. In addition, a threshold of 150 to define Cyclin E1 overexpression was chosen in the present study, since the association of H-score with poor prognosis and/or platinum resistance was reported as significant only for studies with a threshold set at > 100, >140, and > 200, and this was not the case with lower threshold (i.e., > 70) [28]. Our data suggests that it may be relevant to re-biopsy patients at post-PARPi progression to evaluate Cyclin E1 expression. Although conducted on a small subset of six patients, we observed low H-score intratumoral heterogeneity from synchronous but anatomically distinct tumour samples, highlighting the reproducibility of Cyclin E1 IHC analysis. However, caution is recommended when interpreting FISH or IHC after taxane-based chemotherapy, because of the polyploidization phenomenon which is an artefact of taxane effect on chromosome mis-segregation and can lead to false positive calls for amplification [28]. A polyploidization phenomenon was found in 14% of the post-NACT samples herein, which is close to the proportion reported for breast cancers (9%) [28]. This could also lead to a “false” temporary Cyclin E1 overexpression using IHC, as previously described [28].

Before systemic treatment in HGOC, the association between Cyclin E1 overexpression and poor prognostic is well reported and Cyclin E1 overexpression is also considered as a marker of platinum-based chemotherapy resistance [2, 3, 25, 29–35]. Herein, we found that Cyclin E1 overexpression in post-PARPi samples was associated with significantly worst outcomes for patients treated with platinum-based chemotherapy post-PARPi progression. However, the absence of control group (PARPi naive patients) limits the inferential power in the present study, which implies that our results, especially those concerning the OS and the PFS, need to be validated on larger series. Moreover, in the absence of comparative cohort, we cannot draw final conclusions about whether the increase in Cyclin E1 expression relates specifically to PARPi therapy or to relapse post-platinum.

The main limitation of this study is the small size of the cohort. However, the strength is the comprehensive characterisation by NGS, IHC, and FISH of samples at several time-points, and their correlation with clinical data. Another limitation is the heterogeneity of the molecular methods used to define the HRD/HRP status herein, using either MyChoice^®^ CDx panel (Myriad Genetics) or sWGS.

To conclude, Cyclin E1 expression in HGOC increases overtime, especially at the relapse during or following PARPi, and appears to be independent from *CCNE1* amplification. Even in a cohort enriched in HRD+ tumours, around 60% of HGOC exhibit high Cyclin E1 expression post-PARPi and could represent good candidates for novel personalised strategies targeting cell cycle checkpoints and replication stress.

## Supplementary information

Supplementary Information

## Data Availability

The authors are committed to data transparency. The data used in the present study can be accessed either in the Supplementary Information or can be obtained directly from the corresponding author on reasonable requests.
