# Supplementary material for: Changes in Cyclin E1 expression and CCNE1 amplification in high-grade ovarian carcinomas post-PARP inhibitor exposure
Source: Br J Cancer. 2026 May 5;135(3):427–35. doi: 10.1038/s41416-026-03440-y (PMC13373207; doi:10.1038/s41416-026-03440-y)
Supplement: Supplementary file 1 — Supplementary Information [file 41416_2026_3440_MOESM1_ESM.docx]

Changes in Cyclin E1 expression and *CCNE1* amplification in high-grade ovarian carcinomas post-PARP inhibitor exposure

**Supplementary Information**

Supplementary Material 1. Detailed IHC and FISH methods.

Supplementary Figure 1. Positive and negative controls for immunohistochemistry.

Supplementary Figure 2. Positive and negative controls for fluorescent *in situ* hybridization.

Supplementary Figure 3. Stratified survival analyses according to the HRD status and to the prior line of chemotherapy before PARPi therapy.

Supplementary Figure 4. Correlation between mean *CCNE1* copy number and cyclin E1 H-score for diagnostic samples.

Supplementary Figure 5. *JAZF1* fluorescent *in situ* hybridization (polyploidization).

**Supplementary Material 1**. Detailed IHC and FISH methods.

**Cyclin E1 immunohistochemistry (IHC)**

For all sample included and according to the manufacturer's instructions, an anti-Cyclin E1 immunohistochemistry (clone EP126, ref. AC-0120RUO, Cell Marque, Rocklin, CA, USA) was performed, using a Ventana Discovery Ultra automated immunostainer (Roche Diagnostics, Rotkreuz, Switzerland) on 4 µm-thick unstained whole tissue sections. The procedure was performed using the following parameters: the primary antibody (dilution: 1:100) was incubated for 60 minutes at 37°C, the secondary detection was performed using the ultraView Universal DAB Detection Kit (Roche Diagnostics), and the antigen retrieval was performed using the antigen retrieval buffer CC2 pH 6 (Roche Diagnostics). Then, the nuclear Cyclin E1 expression in tumor cells was quantified by two pathologists (A.T. and C.G.) blinded to the clinical and molecular data, outside necrosis or areas with electrocoagulation, on at least 100 tumor cells. Both the proportion of positive tumor cells (%) and the intensity of staining (0, negative staining; 1+, weak staining; 2+, moderate staining; and 3+, strong staining) were considered, using a H-score, defined as a score from 0 to 300, and calculated as follow: H-score = (1×proportion of 1+ tumor cells) + (2×proportion of 2+ tumor cells) + (3×proportion of 3+ tumor cells). The threshold ≥150 was chosen to define overexpression (Cyclin E1 high *versus* Cyclin E1-low). Positive (placenta, HGSOC with known *CCNE1* amplification determined by NGS prior to the present study) and negative (lymph node) controls were used for each run (Supplementary Figure 1).

Of note, for six tumors, the immunohistochemistry was performed on two different paraffin blocks and the H-scores obtained were compared, in order to study the intratumoral (spatial) heterogeneity of Cyclin E1 expression.

***CCNE1* fluorescent *in situ* hybridization (FISH)**

*CCNE1* FISH was performed on post-poly(adenosine diphosphate-ribose) polymerase inhibitor (PARPi) samples to investigate whether the Cyclin E1 overexpression using immunohistochemistry was due to *CCNE1* amplification. FISH were also performed on diagnostic and post-neoadjuvant chemotherapy (NAC) samples to study temporal heterogeneity of *CCNE1* copy number according to treatments. FISH were performed on 4 µm-thick unstained whole tissue sections, using a *CCNE1*-centromere of chromosome 19 (CEN19) FISH probes (dye color: orange [*CCNE1*] and green [CEN19]; Empire Genomics, Buffalo, NY, USA), and the ZytoLight FISH-Tissue Implementation Kit (ZytoVision, Bremerhaven, Germany). Briefly, dewaxed tissue sections were hydrated using successive ethanol baths (85% and then 70%) for 5 minutes each. Tissue sections were then pretreated by incubation in heat pretreatment solution citric (supplied with the kit; antigen retrieval buffer) in a water bath at 98°C for 15 minutes, followed by one to two drops of pepsin solution (supplied with the kit) per tissue section at 37°C for 3 minutes for biopsy samples and 5 minutes for surgical specimens. Then, tissue sections were washed using wash buffer SCC (supplied with the kit) for 5 minutes, then in deionized water for 1 minute, dehydrated using successive ethanol baths (70%, 85%, and 100%) for 1 minute each, and air-dried. For each slide, after DNA denaturation at 83°C for 5 minutes, hybridization of *CCNE1*-CEN19 FISH probes was performed at 37°C for 15-17 hours (10µl for each slide: 3µl of probes, 7µl of dilution buffer). After probe hybridization’s, tissue sections were washed using a stringent wash buffer DAKO 1× (DAKO, Agilent Technologies, Santa-Clara, CA, USA) at room temperature for 5 minutes, then incubated using the same wash buffer at 65°C in a water bath for 2 minutes, and then washed using a wash buffer DAKO 1× (DAKO, Agilent Technologies) at room temperature for 5 minutes. Next, tissue sections were dehydrated using successive ethanol baths (70%, 85%, and 100%) for 1 minute each. Finally, slides were then counterstained with 10 µl of 4’,6-diamino-2-phenylindole in antifade solution (DAPI/Vectashield; VectorLabs; Newark, CA, USA) each.

The interpretation was performed by a pathologist (A.T.) and a cytogeneticist (A.V.). The FISH signals were counted under 100× objective lens. *CCNE1* amplification was defined as ≥8 copies of *CCNE1* per tumor cell. In case of *CCNE1* amplification, the spatial distribution of the signals was also noted, either as double minute-type amplicon (discrete, countable signals), or as homogenously staining region (HSR)-type amplicon (aggregated clusters of signals), or ring-like structures suggesting an amplification within a ring chromosome. Moreover, the intratumoral homogeneity or heterogeneity of the amplification was specified. In the absence of *CCNE1* amplification, the tumors were classified in four other different patterns: i) *CCNE1* gain (*i.e.* *CCNE1*/CEN19 ratio ≥2); ii) high polysomy of chromosome 19 (*i.e.* ≥4 copies of *CCNE1* and CEN19 signals in >40% of cells); iii) polyploidization (*i.e.* high polyploidy in giant tumor cells after taxane-based chemotherapy); and iv) absence of *CCNE1* significant alteration (*i.e.* diploidy, triploidy, or tetraploidy in less than 40% of tumor cell). The *CCNE1* copy number estimation was based: i) either on the visual fluorescence intensity of the cluster as compared to the CEN19 spot; or ii) when possible, was estimated in the nuclei with relaxed DNA where a count of the spots of *CCNE1* was performed. The count of each signal was performed on at least 100 tumor cells in three different areas of the slides, except for post-NAC samples for which the count was performed on at least 25 tumors cells for CRS 3 tumors. In the absence of *CCNE1* significant alteration, CEN19 monosomy, gain (CEN19/*CCNE1* ratio ≥2), or amplification (≥8 spots of CEN19) were also noted. Positive (HGSOC with a known *CCNE1* amplification determined by NGS prior to the present study) and negative (cellblock of SK-OV-3 cell lines [ATCC, Manassas, VA, USA], with a known absence of *CCNE1* amplification) controls were used (Supplementary Figure 2).


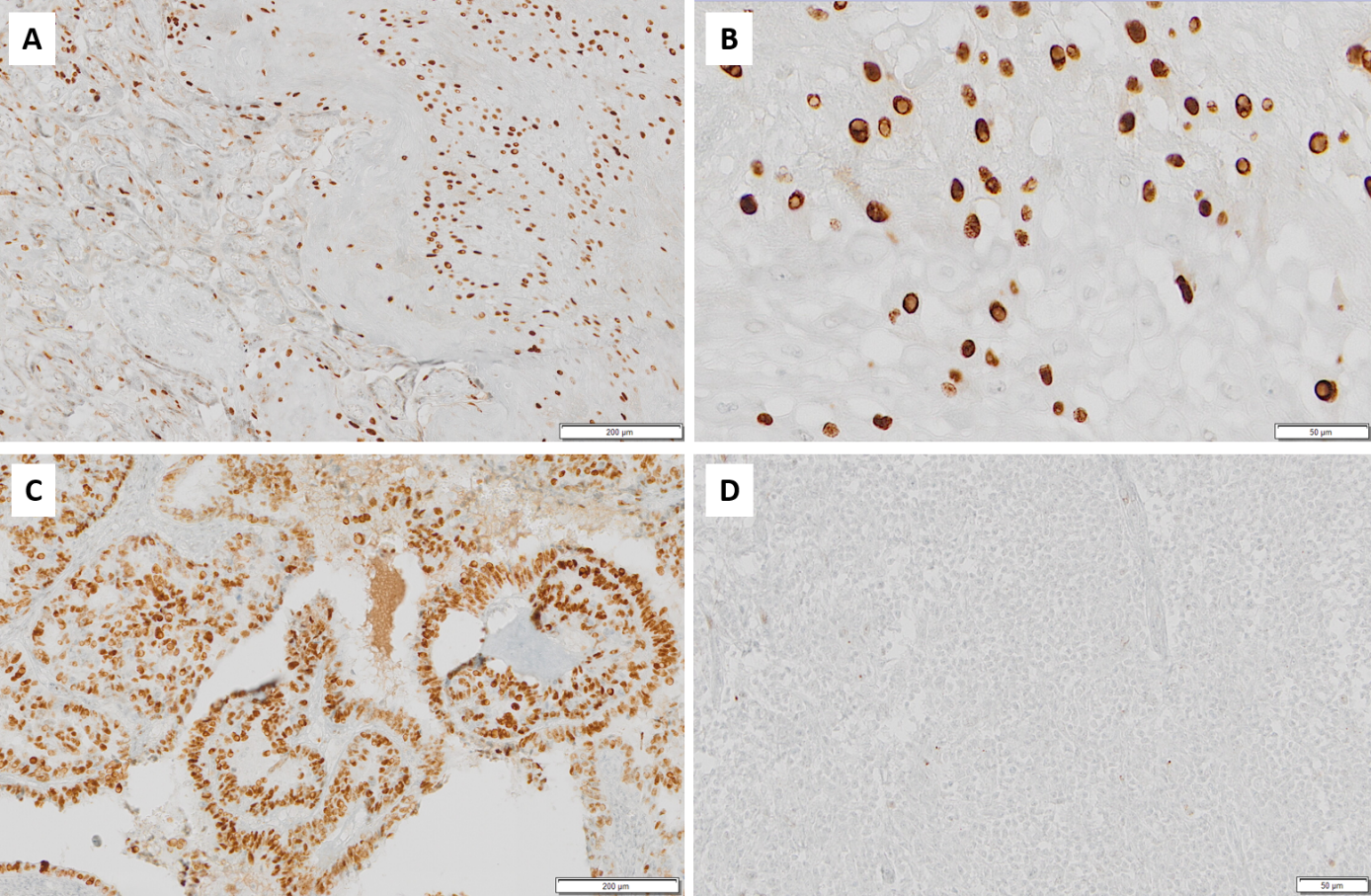


**Supplementary Figure 1**. Positive and negative controls for Cyclin E1 IHC.

A. Positive control (placenta): variable positivity of Cyclin E1 IHC of villous trophoblast (left) and intense positivity of intermediate trophoblast (right) (x70 magnification). B. Positive control (placenta): intense Cyclin E1 positivity of intermediate trophoblast (right) (x200 magnification). C. Positive control (high-grade serous ovarian carcinoma with known *CCNE1* amplification identified by targeted next-generation sequencing prior to the present study): intense Cyclin E1 positivity of tumor cells (x70 magnification). D. Negative control: lymph node parenchyma, absence of Cyclin E1 expression using immunohistochemistry (x170 magnification).

IHC: Immunohistochemistry.


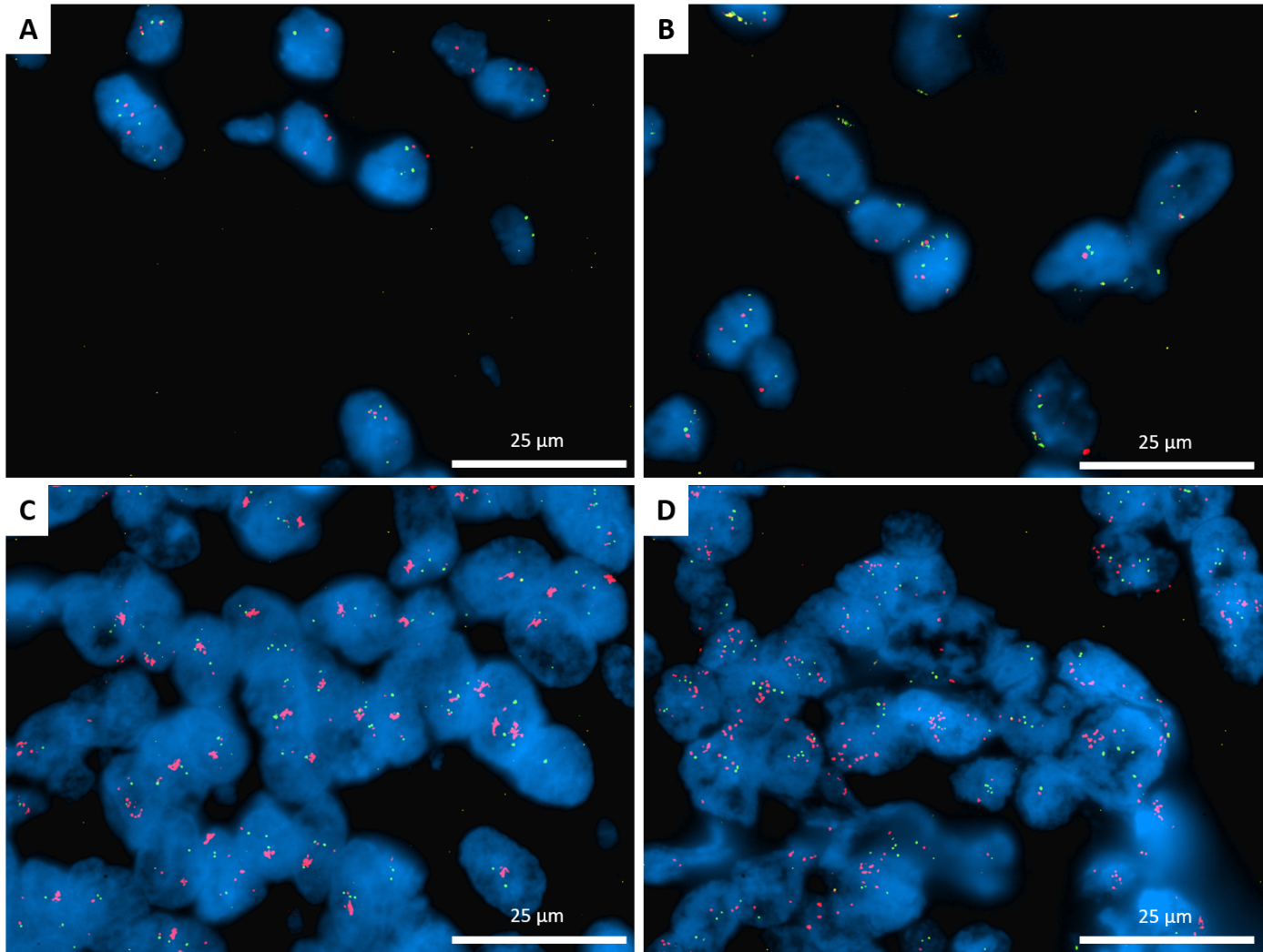


**Supplementary Figure 2**. Positive and negative controls for FISH.

A and B. *CCNE1* (red)–CEN19 (green) FISH showing two or three copies of *CCNE1* and CEN19 per tumor cell (SK-OV-3 cell lines; x1000 magnification). C. *CCNE1* amplification (HSR-type amplicon; x1000 magnification). D. *CCNE1* amplification (double-minute-type amplicon; x1000 magnification).

CEN19: Centromere chromosome 19; FISH: Fluorescent *in situ* hybridization; HSR: homogenously staining region.


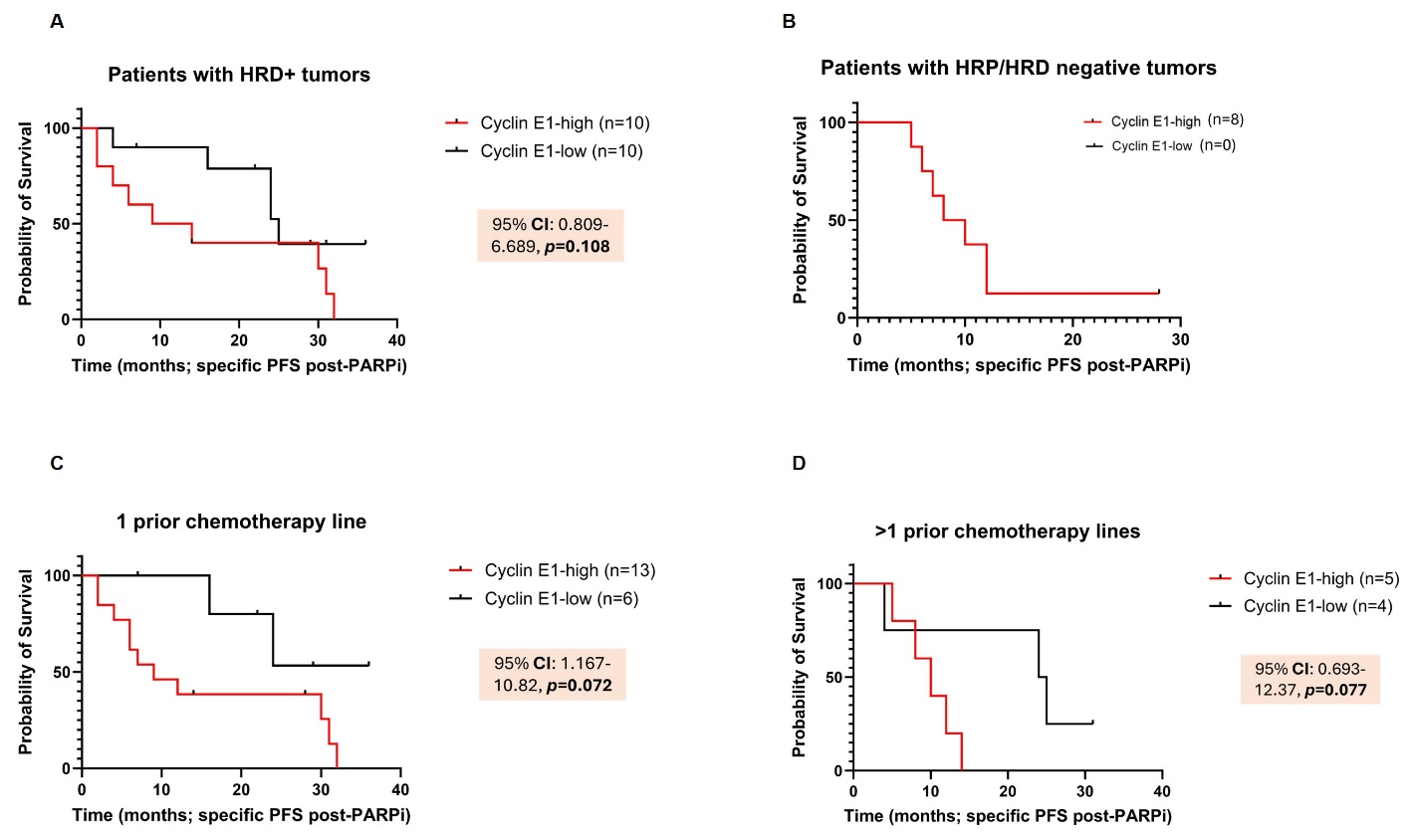


**Supplementary Figure 3**. Stratified survival analyses according to the HRD status and to the prior line of chemotherapy before PARPi therapy.

Overall, no signification modifications in the Kaplan-Meier curve shapes were found when comparing with Figure 2G.

A. Concerning patients with HRD+ tumors treated using platinum-based therapy at the post-PARPi progression, no significant difference in the PFS was found when comparing Cyclin E1-high *versus* Cyclin E1-low tumors (11.5 *versus* 25 months; 95% CI: 0.809-6.689; p=0.108). B. Kaplan-Meier curve of patients with HRP/HRD-negative tumors: all patients included were Cyclin E1-high. C. Concerning patients treated using platinum-based therapy at the post-PARPi progression but who underwent only 1 prior chemotherapy line before the post-PARPi progression, no significant difference in the PFS was found when comparing Cyclin E1-high *versus* Cyclin E1-low tumors (9 months *versus* not reached; 95% CI: 1.167-10.82; p=0.072). D. Concerning patients treated using platinum-based therapy at the post-PARPi progression but who underwent >1 prior chemotherapy line before the post-PARPi progression, no significant difference in the PFS was found when comparing Cyclin E1-high *versus* Cyclin E1-low tumors (10 *versus* 24.5 months; 95% CI: 0.693-12.370; p=0.077).

PARPi: post-poly(adenosine diphosphate-ribose) polymerase inhibitor. HRD+: homologous repair deficiency-positive; HRP/HRD-negative: homologous repair proficiency.


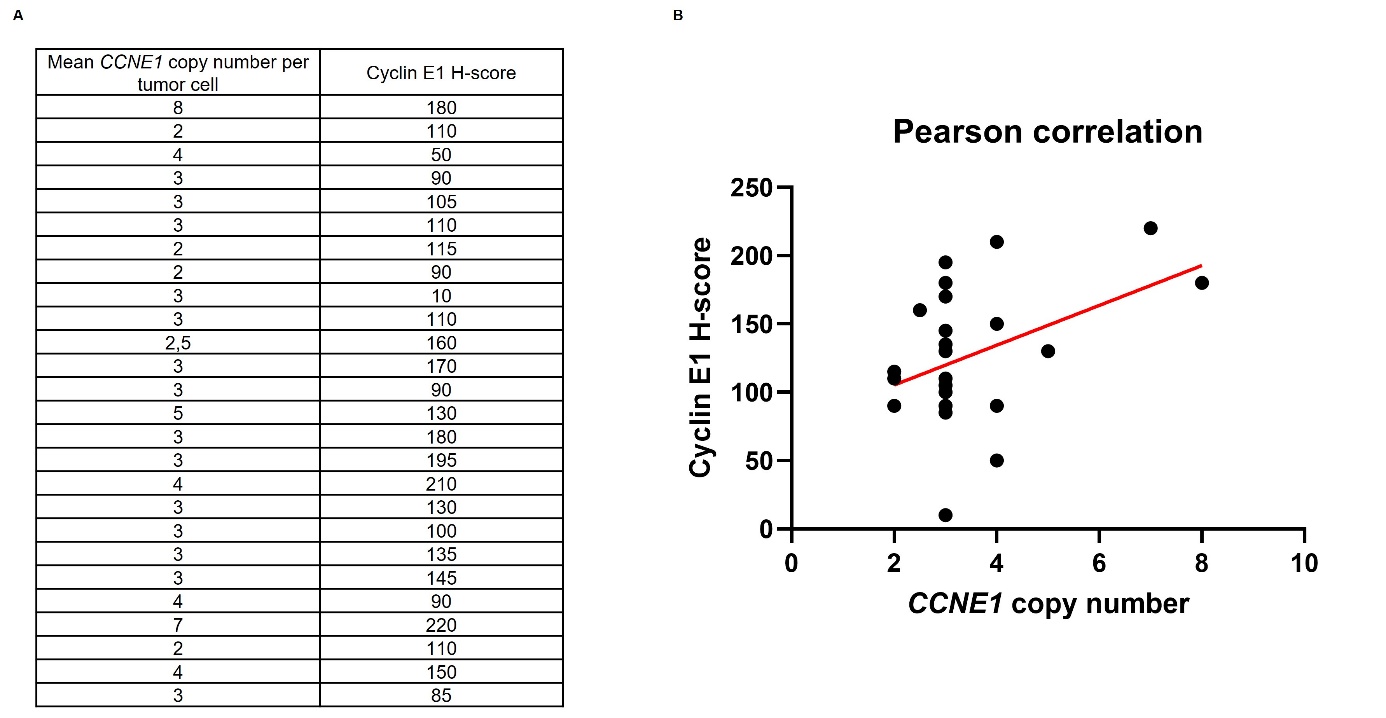


**Supplementary Figure 4.** Correlation between mean *CCNE1* copy number per tumor cell and cyclin E1 H-score for diagnostic samples.

A. Distribution of mean *CCNE1* copy number and cyclin E1 H-score among the 26 diagnostic samples available. B. Pearson correlation: the correlation between cyclin E1 H-score and mean *CCNE1* copy number per tumor cell at diagnosis was moderate (r=0.42; 95% confidence interval [0.039-0.695, p=0.033).


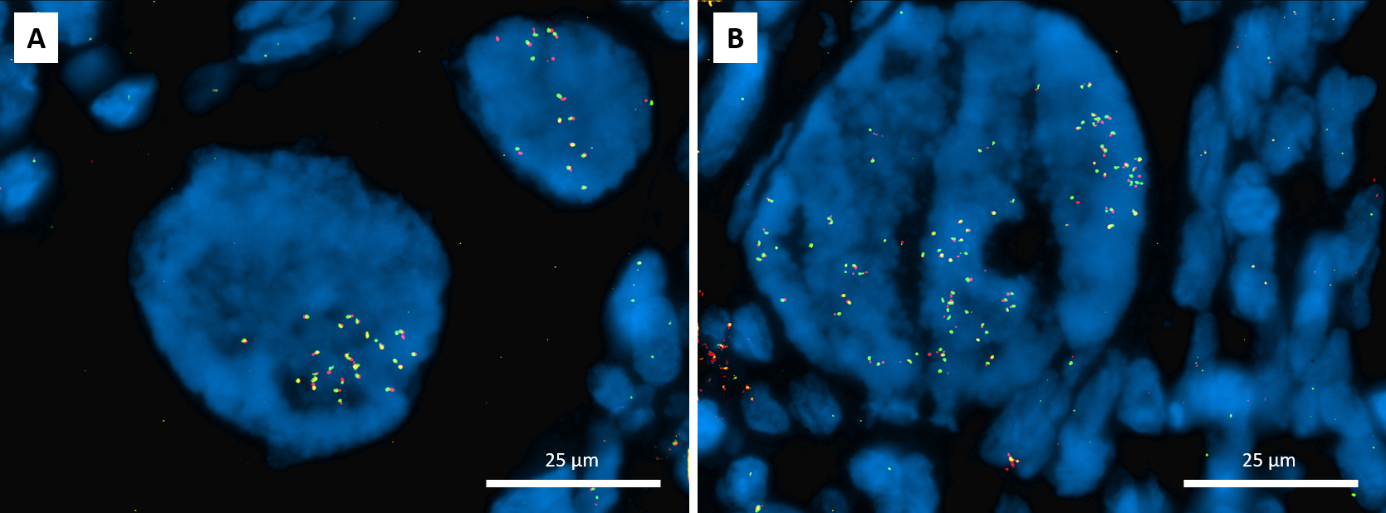


**Supplementary Figure 5**. *JAZF1* fluorescent *in situ* hybridization (polyploidization).

The *JAZF1* probe showed >20 copies of *JAZF1* in some giant and pleomorphic tumor cells after taxane-based chemotherapy, confirming the polyploizidation phenomenon (similar results than when targeting *CCNE1* and CEN19; *JAZF1* is located on chromosome 7; x1000 magnification).

CEN19: Centromere chromosome 19; FISH: Fluorescent *in situ* hybridization.
